# Solar-rechargeable battery based on photoelectrochemical water oxidation: Solar water battery

**DOI:** 10.1038/srep33400

**Published:** 2016-09-15

**Authors:** Gonu Kim, Misol Oh, Yiseul Park

**Affiliations:** 1 Division of Nano and Energy Convergence Research, Deagu Gyeongbuk Institute of Science & Technology (DGIST), 333, Techno Jungang Daero, Hyeonpung-myeon, Dalseong-gun, Daegu 42988, Korea

## Abstract

As an alternative to the photoelectrochemical water splitting for use in the fuel cells used to generate electrical power, this study set out to develop a solar energy rechargeable battery system based on photoelectrochemical water oxidation. We refer to this design as a “solar water battery”. The solar water battery integrates a photoelectrochemical cell and battery into a single device. It uses a water oxidation reaction to simultaneously convert and store solar energy. With the solar water battery, light striking the photoelectrode causes the water to be photo-oxidized, thus charging the battery. During the discharge process, the solar water battery reduces oxygen to water with a high coulombic efficiency (>90%) and a high average output voltage (0.6 V). Because the reduction potential of oxygen is more positive [E^0^ (O_2_/H_2_O) = 1.23 V *vs.* NHE] than common catholytes (e.g., iodide, sulfur), a high discharge voltage is produced. The solar water battery also exhibits a superior storage ability, maintaining 99% of its specific discharge capacitance after 10 h of storage, without any evidence of self-discharge. The optimization of the cell design and configuration, taking the presence of oxygen in the cell into account, was critical to achieving an efficient photocharge/discharge.

To enable the utilization of solar energy as a green and sustainable energy source, hydrogen generation using photoelectrochemical cells (PEC), and the subsequent conversion of the hydrogen gas to electricity using fuel cells, has been extensively developed as a green technology for a future hydrogen-based economy^1^,^2^,^3^,^4^,^5^. The generation of electricity by fuel cells using hydrogen produced by PEC water splitting is a truly environmentally benign technology since solar irradiation provides the energy source, and water is both the initial and final material^6^. However, before the hydrogen can be used in a fuel cell, additional separation and liquefaction processes are required so that the hydrogen can be stored and transported (Fig. 1a)^7^,^8^,^9^.

Therefore, as an alternative technology, a solar-powered electrochemical energy storage (SPEES) system, which integrates a photoelectrochemical cell and an electrochemical cell into a single device, has been investigated as a means of simultaneously converting and storing solar energy^10^. Although the SPEES concept was initially proposed in 1976 by Hodes *et al*. who combined a chalcogenide PEC with redox-based catholytes (electrolyte in the cathode part), to date this concept has not been studied extensively^11^. Most SPEES systems are based on the redox reactions between catholytes (e.g., sulfur, iodide, vanadium) and the anode. Upon being irradiated, photo-generated electron-hole pairs in a photoelectrode (PE) are separated by transferring electrons to the anode through an external circuit, and the holes are injected into the catholyte. Therefore, the theoretical cell voltage is determined by the redox potential difference between the anode and catholyte. For example, Licht reported on a SPEES system configured using n-type Cd (Se_0.65_Te_0.35_), CoS, and Sn/SnS as the photoelectrode (PE), counter electrode (CE), and anode, respectively^12^,^13^. In his design, a polysulfide-based aqueous electrolyte was used as the catholyte (E^0^ (S/S^2−^) = −0.48 V *vs.* NHE), with a redox potential difference with the redox-active anode (SnS) (E^0^ (Sn^2+^/Sn) = −0.95 V *vs.* NHE) of around 0.5 V. As a result, this system produced a cell voltage of around 0.47 V, in the dark, after being photocharged. However, the high fabrication cost and toxicity of the Cd-based photoelectrode and the negative redox potential of the polysulfide-based electrolyte limit further development of this system^10^. A tri-iodide/iodide (I_3_^−^/I^−^) electrolyte [E^0^ (I_3_^−^/I^−^) = 0.5 V in water, 0.35 V in organic solvent (*vs.* NHE)] was also commonly employed as a catholyte in SPEES systems^14^,^15^,^16^. In such systems, dye-sensitized TiO_2_-PE and a platinum-CE and a WO_3_ (or polypyrrole) anode were used. WO_3_ and polypyrrole electrodes store photogenerated electrons by Li^+^ intercalation or ClO_4_^−^ doping, respectively, while I^−^ is oxidized to I_3_^−^ by the photogenerated holes during photocharging. When discharged, I_3_^−^ was reduced again to I^−^ by accepting electrons from the WO_3_ or polypyrrole electrodes. SPEES system replacing the solid anode with a redox-active anolyte (electrolyte in the anode part) species such as [Fe(C_10_H_15_)_2_]^+^/[Fe(C_10_H_15_)_2_], Li_2_WO_4_/Li_2+x_WO_4_ and quinoxaline (C_8_H_6_N_2_/C_8_H_6_N_2_^−^), coupled with the use of a tri-iodide/iodide catholyte was also investigated^17^,^18^,^19^. However, an iodine catholyte has drawbacks such as its relatively low energy storage specific capacity, negative redox potential, visible light absorption, and its overly corrosive nature for practical applications^10^.

Therefore, in this study, we set out to develop an environmental friendly “solar water battery” based on a water oxidation reaction instead of the redox chemistry of a catholyte. Such a solar water battery incorporates the advantages of both the solar water splitting techniques and previously developed SPEES systems: environmental friendliness and the simultaneous conversion and storage of solar energy. In a solar water battery, the catholyte is replaced with water which is oxidized by the photoelectrode during photocharging, and oxygen is reduced to water during discharge. Therefore, the solar water battery does not use or produce any harmful materials. And a high discharge voltage can be produced, because the reduction potential of oxygen is more positive [E^0^ (O_2_/H_2_O) = 1.23 V vs. NHE] than common catholytes (e.g., iodide, sulfur). Furthermore, the solar water battery can both convert solar energy to electricity and simultaneously store it in a single device in the same way as in previous SPEES systems (Fig. 1b). We designed a new configuration for a solar battery to operate it successfully with a high coulombic efficiency. Also, we investigated the important factors affecting the efficiency of the solar water battery and discuss how it operates.

## Results

### Configuration and mechanism of solar water battery

**Figure 2:**
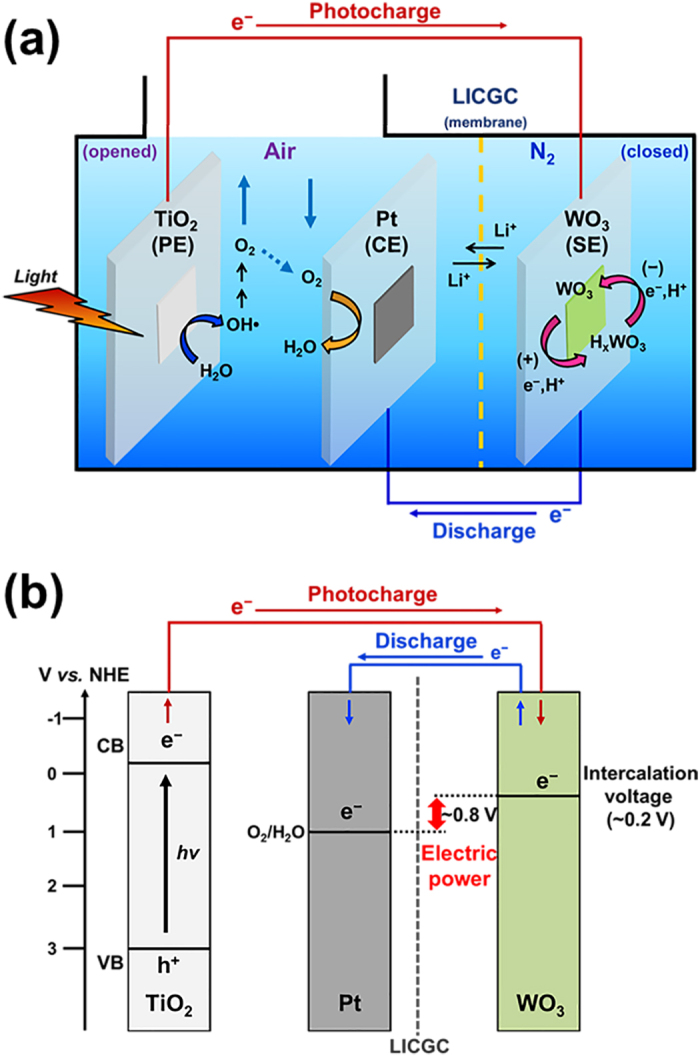
(**a**) Photocharging and discharging mechanism in solar water battery, (**b**) energy diagram for each electrode (TiO_2_ photoelectrode (PE), Pt counter electrode (CE), WO_3_ storage electrode (SE)).

The cell configuration of the solar water battery is shown in Fig. 2a. The solar water battery has three electrodes: a photoelectrode (PE), electron storage electrode (SE), and counter electrode (CE). For the PE, n-type semiconductor TiO_2_ was used as this offers a strong oxidizing power and superior stability. For the SE, WO_3_ with an intercalation potential of around 0–0.4 V (*vs.* NHE), which is comparable to the proton reduction potential, was used. For the CE, Pt nanoparticles coated FTO electrode was used to increase the oxygen reduction reaction by providing a greater contact area (see Methods).

The morphology and crystallinity of each electrode materials were characterized and confirmed by FE-SEM imaging and XRD measurements (Supplementary Fig. 1). The atmospheres in the solar water battery were divided to improve the efficiency of the photocharge/discharge. The SE is kept in an anoxic state to prevent the self-discharge which is induced by the oxygen reduction reaction. Meanwhile, the part containing PE and CE is opened to air to offer the oxygen efficiently for the reduction on CE and to prevent a volume expansion of cell by the produced oxygen from water oxidation on PE. The detailed reasons and the experiment results for using the different atmosphere will be discussed later. To establish the different atmospheres in each part, a lithium ion conduction glass ceramic (LICGC) membrane, which allows only the transport of lithium ions, but not other ions or oxygen, is applied.

The proposed working mechanism of the solar water battery is shown in Fig. 2b. When illuminated by sunlight, the TiO_2_-PE is excited and generates an electron-hole pair. The photogenerated electrons in the TiO_2_-PE are transferred to the WO_3_-SE through an external circuit under the influence of a thermodynamic energy level difference between the TiO_2_ and WO_3_. The transferred electrons are stored in the WO_3_-SE by redox reactions between W^6+^ and W^5+^, accompanied by H^+^ ion insertion (WO_3_ + *x*e^−^ + *x*H^+^ ↔ H_x_WO_3_). During the discharge process, WO_3_-SE and Pt-CE are connected and the electrons stored in the WO_3_-SE move back to the Pt-CE, thus producing electric power. Theoretically, our solar water battery could produce an output voltage of around 0.8 V, which is the difference between the oxygen reduction potential (1.03 V *vs.* NHE (at pH 3)) at the Pt-CE and the H^+^ ions-intercalation potential of the WO_3_-SE (~0.2 V vs. NHE (at pH 3)) (Fig. 2b). We investigated, separately, the photocharge, storage and discharge processes occurring in the solar water battery, and discussed how the solar water battery operates in each process, thus providing evidence for each of those processes.

### Solar-energy conversion in TiO_2_ electrode

**Figure 3:**
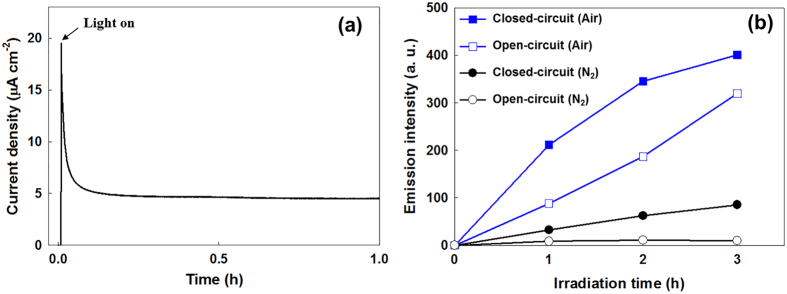
Time profile of (**a**) photocurrent and (**b**) coumarin-OH adduct (7-hydroxycoumarin) generation on photoelectrode when irradiated with sunlight (coumarin 1 mM; pH 3.0).

We first checked the feasibility of using WO_3_-SE as an electrochemical storage electrode in a 2-electrode system (Pt counter electrode) by performing galvanostatic charge/discharge in the dark with a current density of 2 mA g^−1^ and a cutoff voltage of −1.1 V (Supplementary Fig. 2). The cell stabilized after 10 cycles and exhibited a coulombic efficiency in excess of 90% over 50 cycles, thus confirming the electron storage ability of the WO_3_ and the well-established cell conditions. Prior to the solar-energy storage test, the TiO_2_-PE was pre-illuminated with sunlight for 16 h under an open-circuit condition to ensure the a removal of all contamination with organic impurities on the TiO_2_ surface^20^. After the cleaning, the TiO_2_-PE was connected to the WO_3_-SE through an external circuit and irradiated with sunlight to instigate the photocharging. Figure 3a shows the electron flow from the TiO_2_-PE to the WO_3_-SE, which was monitored without applying any external bias. When the TiO_2_-PE is illuminated, a continuous and stable photocurrent is generated. This is clear evidence for the photo-generated electrons’ transfer from the TiO_2_-PE to the WO_3_-SE.

The force driving the electron transfer from the photoexcited-TiO_2_ to the WO_3_ is the difference between the Fermi level of the photoexcited-TiO_2_ and that of WO_3_ (ΔV (V_F, TiO2_ − V_F, WO3_)). This continues until the energy levels of both electrodes are the same. We estimated the flat band potential of the WO_3_-SE before and after the photocharge according to the Mott-Schottky plot (Supplementary Fig. 3). The flat band potential of WO_3_-SE was around 0.1 V (*vs.* NHE) before the photocharge, becoming –0.3 V (*vs.* NHE) after being photocharged for 16 h. The shift of the flat-band potential to the negative direction supports our claims that the electrons are transferred to WO_3_-SE and accumulated. It is important to note that the flat band potential obtained by the Mott-Schottky plots may have to be treated with caution due to the polycrystalline nature of the film, therefore, the estimated values might be different with the real values. But, the shift of the flat band potential is reliable to support the electron storage in WO_3_-SE. To confirm whether the electron transfer is a result of the photoelectrochemical oxidation of the water on the TiO_2_-PE, the possible products (OH•, H_2_O_2_, O_2_) of water oxidation were measured. (Equation 1, 2, 3).


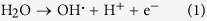



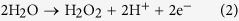



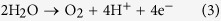


Firstly, the OH radicals which appear as intermediaries during the water oxidation were measured using coumarin reagent. The coumarin traps the OH radical in the electrolyte, thus forming fluorescent 7-hydroxycoumarin^21^. Therefore, the amount of OH radicals could be quantified by measuring the fluorescence emission intensity of the 7-hydroxycoumarin. Figure 3b shows the time profiles of the 7-hydroxycoumarin generation under different experimental conditions during photoelectrochemical water oxidation by the TiO_2_-PE. When the TiO_2_-PE is connected to the WO_3_-SE (closed circuit), the fluorescence emission intensity of the 7-hydroxycoumarin was proportional to the duration of the irradiation, indicating OH radical generation by photoelectrochemical water oxidation. However, the OH radicals were also produced on the TiO_2_-PE without any connection to the WO_3_-SE (open circuit) in a system, which can be attributed to the photocatalytic oxidation of the water on the TiO_2_-PE. Because the photogenerated electrons on the TiO_2_-PE are consumed by the dissolved oxygen, the photogenerated holes can be used for the water oxidation. However, the quantity of OH radicals produced with the open circuit was less than that produced with the closed circuit. This implies that the WO_3_-SE connection (closed circuit) encourages the separation of the e^−^/h^+^ pair during the photocharging process by providing a greater driving force for the transfer of the photogenerated electrons to the WO_3_-SE, leading to the increased oxidation of the water. To clarify this, the same experiments were carried out under anoxic (N_2_) conditions. Under anoxic conditions, almost no OH radical generation was observed with the open circuit due to the fast e^−^/h^+^ pair recombination in the absence of oxygen. However, when the WO_3_-SE acts as an electron acceptor and is connected (closed circuit), a significant quantity of OH radicals was clearly generated. These results confirm that the WO_3_-SE can provide the driving force needed to transfer the photogenerated electrons, and that photoelectrochemical water oxidation occurs on the TiO_2_-PE without an external bias. Our results prove that the water acts as the electron source to scavenge the hole in the TiO_2_-PE. The generation of H_2_O_2_ which can be produced by two-electron transfer reaction of water was also measured indicating the water oxidation during the photocharging is evident (Supplementary Fig. 4). The concentration of H_2_O_2_ increased linearly during 24 h of photocharging. Since the H_2_O_2_ is easily decomposed in the presence of Pt catalyst, the measurement of H_2_O_2_ was carried out in the absence of Pt-CE. Besides, the decomposition of H_2_O_2_ (2H_2_O_2 _→ 2H_2_O + O_2_) in the presence of Pt-CE after the photocharging was also observed in our experiment results as shown in Fig. S4. After measurement of H_2_O_2_ during the photocharging in the absence of Pt electrode for 24 h, the resultant electrolyte was transferred into two vials. Pt electrode was immersed into the electrolyte in one vial, and another vial was kept without Pt electrode. The concentrations of H_2_O_2_ in the both electrolytes were compared after 6 h. The sample without Pt electrode showed a small change of the H_2_O_2_ concentration, on the other hand, that with Pt electrode showed 100% decomposition of H_2_O_2_. These results show us that O_2_ can be produced through the H_2_O_2_ decomposition during the photocharging and subsequently it can be used for the reduction of oxygen during the discharging on Pt-CE. O_2_ also can be generated by the four-electron transfer from water. However, when we detected O_2_ evolution in anoxic condition, the produced amount of O_2_ after 24 h of photocharging was very small or almost negligible. It is attributed to the low efficiency of O_2_ production by both pathways (H_2_O_2_ decomposition, water oxidation) due to a low solar energy conversion efficiency of TiO_2_ under 1 sun condition, and a low catalytic activity for water oxidation of TiO_2_-PE without oxygen evolution catalysts.

### Storage of photo-generated electrons in WO_3_ electrode

**Figure 4:**
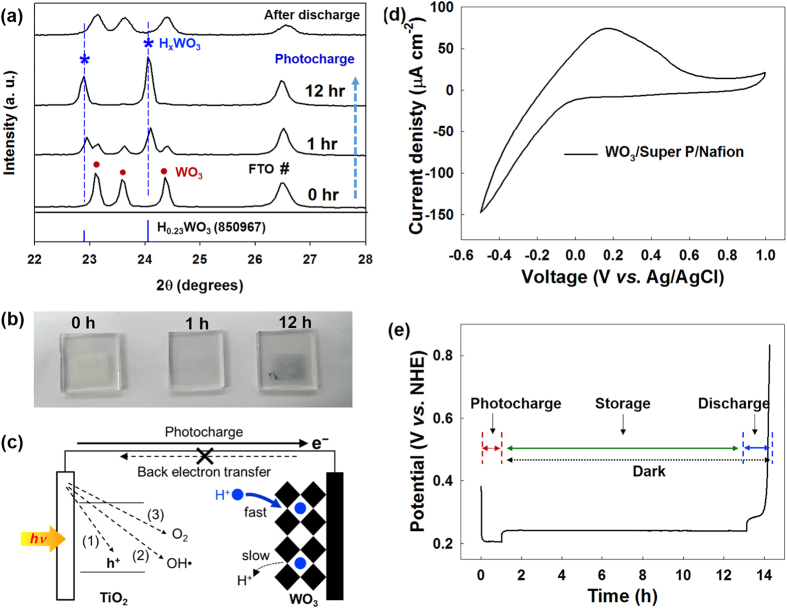
(**a**) Phase change in XRD patterns of WO_3_ electrode and (**b**) Color change in WO_3_ electrode before and after photocharging (0–12 h). (**c**) Possible pathways for back electron transfer during photocharging. (**d**) CV curves of WO_3_ electrode at a scan rate of 10 mV s^−1^. (**e**) Monitoring of electrical potential of WO_3_ electrode during photocharging, storage, and discharge.

When the photogenerated electrons are transferred to the WO_3_-SE, tungsten bronze (H_x_WO_3_) is formed by H^+^ ion insertion (WO_3 _+ *x*e^−^ + *x*H^+^ ↔ H_x_WO_3_). The formation of H_x_WO_3_ is confirmed by X-ray diffraction (XRD) measurement, as shown in Fig. 4a. Compared to the XRD pattern of the WO_3_ before being irradiated with sunlight, new peaks appear after 1 h of photocharging. The intensities of the new peaks become more obvious after the photocharging continues for another 12 h, while the original peaks of the WO_3_ almost disappear. The new peaks can be indexed as peaks generated by H_x_WO_3_ (refer to JCPDS card: no. 85-0967), indicating the conversion of the WO_3_ to H_x_WO_3_ by the transfer of electrons from the TiO_2_-PE during photocharging^22^. The conversion of the WO_3_ to H_x_WO_3_ can also be confirmed by observing the color change during photocharging, as shown in Fig. 4b. For this experiment, a WO_3_ electrode was prepared without any conductive carbon. (The carbon would interfere with the observation of the color.) The color of the WO_3_ electrode was found to change from pale yellow to blue, which is the color of H_x_WO_3_, as the photocharge progressed, again pointing to the storage of electrons in the WO_3_, thus forming H_x_WO_3_.

Figure 4c illustrates various possible back electron transfer pathways for the electrons stored in the WO_3_-SE to the TiO_2_-PE during the photocharge. The stored electrons in the WO_3_-SE can go back to (1) recombine with the hole in the TiO_2_-PE or (2) react with OH radical or (3) react with the oxygen supplied from the air. However, the stable intercalated form of the H_x_WO_3_ suppresses the back electron transfer during the photocharge. To verify this, we have measured cyclic voltammetry (CV) of WO_3_-SE. As shown in Fig. 4d, a sharp peak in the current appeared during cathodic polarization, while a moderate and broad curve was observed during anodic polarization. The CV results indicate that the WO_3_ has fast intercalation but slow deintercalation characteristics, and that it suppresses the back electron transfer from the H_x_WO_3_ to the TiO_2_ during the photocharging process. This proves that the WO_3_ has excellent storage properties as the SE of the solar water battery, which is needed to overcome the attraction of the strong oxidants, namely, the holes in PE, the OH radicals, and the oxygen. The stable electron storage in the WO_3_ was also identified by the monitoring of the electrical potential of the WO_3_-SE during photocharge/storage/discharge, as shown in Fig. 4e. When the TiO_2_-PE was irradiated with sunlight for the photocharging, the electrical potential of the WO_3_-SE (a mixed potential of TiO_2_-PE and the WO_3_-SE owing to an ohmic contact of both electrodes) was decreased sharply and saturated at around 0.2 V (*vs.* NHE). When the light irradiation was closed and both electrodes were disconnected to halt the photocharging, the electrical potential of the WO_3_-SE (a single potential of the WO_3_-SE) increased slightly to 0.23 V (*vs.* NHE). This correspond to the intercalation potential of the H^+^ into the WO_3_^23^,^24^. The electrical potential of the WO_3_-SE in an open-circuit condition was well maintained for 10 h, thus exhibiting stable electron storage without self-discharge. In addition to strong storage properties of WO_3_-SE, the anoxic state of the WO_3_-SE part in the solar water battery contributes to the stable storage without self-discharge by preventing the reaction of the electrons with the dissolved oxygen in the electrolyte during the storage period.

### Discharge generating electric power

**Figure 5:**
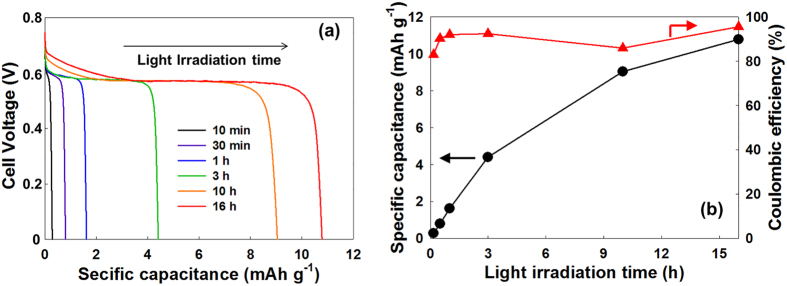
(**a**) Galvanostatic discharge voltage profile and (**b**) specific discharge capacitance and coulombic efficiency after photocharging for 10 min–16 h. Discharge current density was 1 mA g^−1^.

To generate electrical power, the photocharged WO_3_-SE were connected to the Pt-CE to discharge, and were galvanostatically discharged with a current density of 1 mA g^−1^ until the cell voltage reached 0 V. According to the voltage profile observed during the discharge in Fig. 5a, this solar water battery produced an stable output voltage of around 0.6 V with a wide voltage plateau, regardless of the duration of the light irradiation. The voltage plateau region is attributed to the H^+^ ion deintercalation process as a faradaic process. This discharge voltage profile proves that this solar water battery can convert solar energy to electricity with a stable output voltage. However, the observed average output voltage (around 0.6 V) is lower than the theoretical output voltage (around 0.8 V), which could be attributed to the overpotential of the deintercalation reaction at the WO_3_-SE and the low ion conductivity of the LICGC membrane. Figure 5b shows the dependence of the discharge capacitance on the duration of the light irradiation. The specific discharge capacitance increased almost proportionally to the duration of the light irradiation, and it confirms again the solar-energy storage in this solar water battery system. Coulombic efficiency of the solar water battery excessed 90% regardless of the duration of the photocharging, and the specific discharge capacitance reached around 10 mAh g^−1^ after 16 h of irradiation. The coulombic efficiency was calculated from the ratio of the total charge of the photocurrent to that of the discharge current. This high coulombic efficiency of the solar water battery points to all the stored electrons in the WO_3_-SE being effectively used to generate electricity. And the reconversion of H_x_WO_3_ to WO_3_ after the discharging was confirmed by XRD as shown in Fig. 4a.

## Discussion

Compared with previous SPEES systems, the solar water battery required a newly designed cell configuration based on the presence of oxygen in the electrolyte. Firstly, the air-atmosphere (opened) condition of the chamber where the TiO_2_-PE and Pt-CE are immersed in is beneficial not only for preventing the expansion of the cell by the generated oxygen during the photocharge, but also for the efficient extraction of electrons from the photocharged WO_3_-SE to Pt-CE. When the Pt-CE chamber is purged with N_2_, to probe the role of the air-atmosphere condition, the coulombic efficiency of the discharge after 1 h of photocharging was drastically reduced to 23% of the value measured in an air-atmosphere condition (92%) (Supplementary Fig. 5). The oxygen produced as a result of the water oxidation during the photocharging could subsequently be used for the reduction on the Pt-CE during the discharge step with regenerating the water in the same chamber (Equation 4).


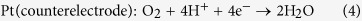


However, the amount of oxygen produced by the TiO_2_-PE is too small to extract the stored electrons in WO_3_ efficiently during the discharging resulting in a low coulombic efficiency. Even if the photoanode can achieve the efficient oxygen evolution by adapting cocatalysts or by using efficient materials for the solar energy conversion, a cell volume expansion by oxygen evolution will be a potential problem for the battery safety. Therefore, we suggested an air-opened cell for the TiO_2_-PE, Pt-CE part for the efficient electrons extraction during the discharging and the safety issue.

**Figure 6:**
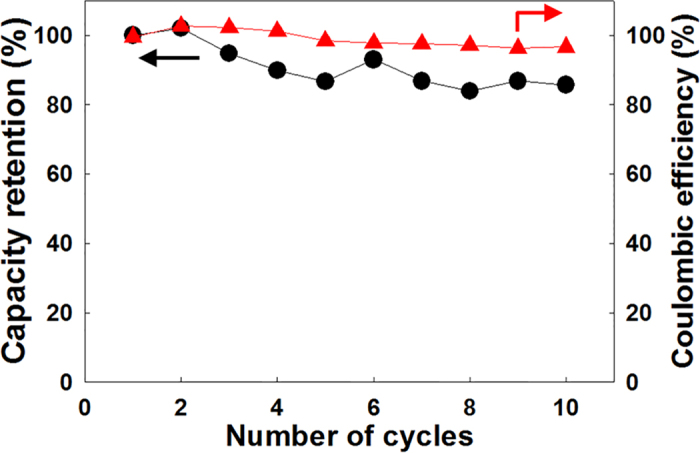
Photocharge/discharge cycle performance plot of solar water battery. (Photocharging time: 1 h, discharge current density: 1 mA g^−1^).

Secondly, WO_3_-SE kept in anoxic condition is essential for stable storage of stored electrons. When we maintained the solar water battery in the open-circuit state for 10 h after 1 h of photocharging, the specific discharge capacitance was found to be 99% of that measured immediately after photocharging (without any storage time). When the entire cell was in the oxic state, a self-discharge occurred and the storage capacity of the cell was severely compromised. On the other hand, when the entire cell was in the anoxic state, photocharging was favored but the discharge efficiency was drastically decreased as we showed previously. The high coulombic efficiency of the solar water battery is clearly related to the configuration of the cell with separate oxic and anoxic chambers. Furthermore, when we tested the cyclability of the solar water battery, we found that the capacity retention rate was 85% after 10 photocharge/discharge cycles while maintaining a high coulombic efficiency (Fig. 6). These results prove that the solar water battery offers a repeatable and promising means of solar energy conversion and storage.

A solar water battery has a merit of relatively high output voltage. For example, there have been some SPEES systems that utilized a dye-sensitized TiO_2_ electrode and WO_3_ electrode as the PE and SE, respectively. These produced a small average output voltage (around 0.4 V) resulted from the difference in the WO_3_ energy levels and the redox potential of I_3_^−^/I^−^ (0.536 V *vs*. NHE) on the counter electrode (Pt)^25^. Meanwhile, because the output voltage of this solar water battery is based on the difference in the WO_3_ intercalation energy level (average 0.2 V *vs.* NHE) and the oxygen reduction reaction [O_2_/H_2_O (1.03 V *vs.* NHE at pH 3], it can have a cell voltage (~0.8 V) that is around 0.4 V larger than the cases described above. Although the maximum discharge voltage of around 0.8 V was not achieved at this point, due to the large overpotential, the development of the counter electrode with a lower overpotential or a thinner LICGC membrane with a higher ionic conductivity could increase the output voltage. The capacity of the solar water battery could also be improved by using more efficient PE, SE, and CE materials which offers better efficiencies. For PE, the water oxidation could be enhanced by using photoactive materials which have a high visible light absorption and a high conduction band edge potential to increase a potential difference (internal bias) with WO_3_, or by adapting an efficient water oxidation cocatalyst to decrease an overpotential for the water oxidation. And for the efficient electron storage, SE could be modified to have a high surface area or good conductivity. The improvement of the efficiency of the oxygen reduction on CE is also could be achieved by the modification of CE property. Recent well-developed and advanced materials for solar water splitting and electrochemical cell (e.g. fuel cell) offer considerable potentials for the development of a solar water battery.

Additionally, a solar water battery has another unique and promising advantage. That is, pollutants can be used to take the place of the water, and be used as an electron source for scavenging photo-generated holes in the PE during photocharging. The system in which the PE chamber is open to the air is better suited to such oxidative degradation of pollutants. We have conducted preliminary tests on the degradation of 4-chlorophenol (a non-biodegradable pollutant), and found that the photocurrent generation was sharply increased and maintained without any drastic decay when 4-chlorophenol was injected into the electrolyte (Supplementary Fig. 6). This indicates that 4-chlorophenol could act as a more efficient oxidant than water, given that simultaneous pollutant degradation and electron storage are feasible in this system. The degradation of organic pollutants is also possible through reductive reaction pathways (i.e. generation of reactive oxygen species (ROS) such as superoxide radical) on the CE during discharge, extending the applications for the treatment of organic pollutants in the night by using the stored electrons during the daytime. More detailed research addressing the development of such a pollutant-degrading solar water battery is currently under investigation.

In conclusion, we have reported on a suitable cell design and configuration for a solar water battery based on water oxidation with a high coulombic efficiency. The solar water battery is environmentally friendly and can provide an alternative to conventional photoelectrochemical water splitting. It could simultaneously convert and store solar energy in a compact system as an alternative to the hydrogen gas generation from photoelectrochemical water splitting, thus simplifying the conventional process from hydrogen production to electricity generation. The cell configuration and operating conditions, such as the atmosphere in each chamber, were critical to the operation of the solar water battery with a good coulombic efficiency. Although the specific discharge capacitance of the cell was not so high (around 10 mAh g^−1^ after 16 h of photocharging) at this point, there is considerable scope for improvement by adapting the numerous materials and technologies developed for solar water splitting which have proven to be an efficient means of solar energy conversion.

## Methods

### Fabrication of solar water battery

The solar water battery consists of a TiO_2_ (P25, Degussa) photoelectrode (PE), a WO_3_ (Aldrich) storage electrode (SE), a platinum (Aldrich) counter electrode (CE), and a lithium-ion-conducting glass ceramic (LICGC, 0.18-mm, Li_1+x+y_Al_x_Ti_2−x_Si_y_P_3−y_O_12_ (OHARA Inc., Japan)) membrane. To fabricate the WO_3_-SE, WO_3_ was mixed with Super P and Nafion (Aldrich) (8:1:1 by weight) in ethanol. The slurry was coated onto an FTO by using a doctor-blade method and then dried at 110 °C in a vacuum oven for 12 h. The TiO_2_-PE and Pt-CE were fabricated using a doctor blade method without any conductive agent or binder as these can possibly affect the light absorption or the surface reaction with the water. TiO_2_-PE and Pt-CE electrodes were prepared according to the literature method^26^. Briefly, TiO_2_ or Pt powder was mixed with polyethylene glycol (PEG). The slurry was cast onto an FTO plate using a doctor-blade technique and then the TiO_2_/FTO electrode or Pt/FTO electrode was heated at 450 °C for 30 min to drive off the binder. When a WO_3_ electrode without any conductive agent or binder was needed, it was prepared using the same method as that for the TiO_2_ electrode or Pt electrode, with the exception of the loading material. The electrolyte used for the solar water battery was 10 mM Li_2_SO_4_ with a pH of 3. The pH of the solution was adjusted to 3.0 by adding either H_2_SO_4_ or NaOH. The deionized water was ultrapure (18 MΩ•cm), prepared using a RephiLe Bioscience purification system.

### Material characterization and analysis

The morphologies of TiO_2_, WO_3_, and Pt were characterized by using field emission scanning electron microscopy (FE-SEM, Hitachi, S-4800). X-ray diffraction (XRD) measurement was carried out using Cu Kα radiation (Empyrean X-ray diffractometer). The generation of OH radicals was measured by using a fluorescence method with coumarin as a chemical trap for the OH radicals (Equation 5). The fluorescence emission intensity of the 7-hydroxycoumarin was measured using a spectrofluorometer (Perkin Elmer LS-55) at 460 nm while being excited at 332 nm.


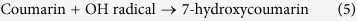


The concentration of H_2_O_2_ at various time intervals was determined by the DPD colorimetric method, which is based on the horseradish peroxidase (Aldrich)-catalyzed reaction of H_2_O_2_ with N,N-diethyl-p-phenylenediamine (DPD, 97%, Aldrich), using a UV-visible spectrophotometer (Simadzu, λ_max_ = 551 nm, ε = 21000 M^−1 ^cm^−1^)^27^.

### (Photo)electrochemical tests

The (photo)electrochemical measurements were carried out using a potentiostat (ZIVE MP1, WONATECH). The WO_3_-SE part of the solar water battery was purged with nitrogen gas prior to the measurement. For the photocharging test, a solar simulator (1 sun, AM 1.5 G, Oriel) was used as the light source. The photocurrent was measured by using the TiO_2_ electrode as a working electrode (WE) and the WO_3_ electrode as both a counter electrode (CE) and reference electrode (RE) with no bias applied. During the discharge, the solar water battery was discharged to 0 V (*vs.* the Pt electrode) using the WO_3_ electrode as the WE and the Pt electrode as the CE and RE at a current density of 1 mA g^−1^. Cyclic voltammetry (CV) measurement was performed by using a conventional three-electrode system in a beaker cell containing the same electrolyte solution under an air atmosphere. CV was carried out over a potential range of −0.5–1 V (*vs*. Ag/AgCl) at a scan rate of 10 mV s^−1^. When the photocharging test using a pollutant as the electron source was conducted, 4-Chlorophenol (4-CP, Aldrich) was instantly injected into the cathode part of the solar water battery during the photocharging such that the final concentration of the 4-CP was 100 μM.

## Additional Information

**How to cite this article**: Kim, G. *et al*. Solar-rechargeable battery based on photoelectrochemical water oxidation: Solar water battery. *Sci. Rep.* **6**, 33400; doi: 10.1038/srep33400 (2016).

## Supplementary Material

Supplementary Information

## Figures and Tables

**Figure 1:**
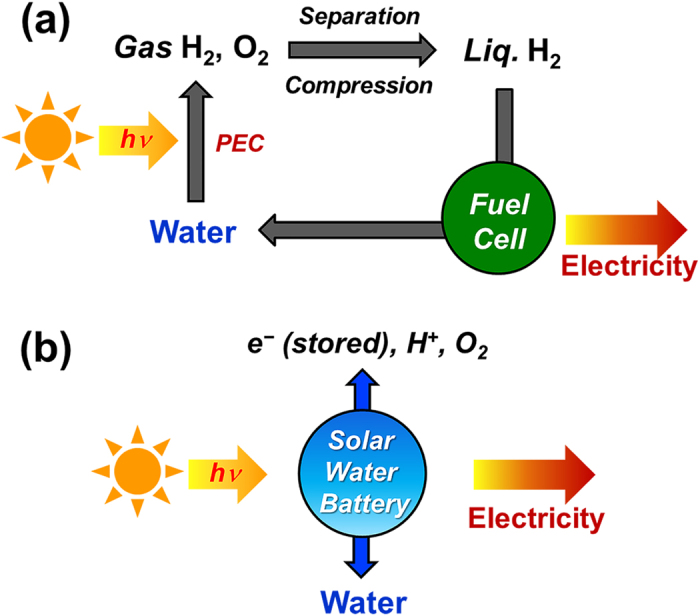
Comparison of electricity generation in (**a**) conventional system combining photoelectrochemical cell and fuel cell and in (**b**) solar water battery.

## References

[b1] Seo J. . Mg-Zr cosubstituted Ta_3_N_5_ photoanode for lower-onset-potential solar-driven photoelectrochemical water splitting. J. Am. Chem. Soc. 137, 12780–12783 (2015). 26426439 10.1021/jacs.5b08329

[b2] Zhang Z. . Toward efficient photoelectrochemical water-splitting by using screw-like SnO_2_ nanostructures as photoanode after being decorated with CdS quantum dots. Nano Energy 19, 318–327 (2016).

[b3] Sheng W. . Quantum dot-sensitized hierarchical micro/nanowire architecture for photoelectrochemical water splitting. ACS Nano 8, 7163–7169 (2014). 24941287 10.1021/nn502121t

[b4] Srivastava S. . Size-selected TiO_2_ nanocluster catalysts for efficient photoelectrochemical water splitting. ACS Nano 8, 11891–11898 (2014). 25365773 10.1021/nn505705a

[b5] Fujishima A. & Honda K. Electrochemical photolysis of water at a semiconductor electrode. Nature 238, 37–38 (1972). 12635268 10.1038/238037a0

[b6] Yang X. . Nitrogen-doped ZnO nanowire arrays for photoelectrochemical water splitting. Nano Lett. 9, 2331–2336 (2009). 19449878 10.1021/nl900772q

[b7] Torella J. P. . Efficient solar-to-fuels production from a hybrid microbial-water-splitting catalyst system. Proc. Natl. Acad. Sci. 112, 2337–2342 (2015). 25675518 10.1073/pnas.1424872112 PMC4345567

[b8] Cipriani G. . Perspective on hydrogen energy carrier and its automotive applications. Int. J. Hydrogen Energy 39, 8482–8494 (2014).

[b9] Zhou L. Progress and problems in hydrogen storage methods. Renew. Sust. Energ. Rev. 9, 395–408 (2005).

[b10] Yu M. . Solar-powered electrochemical energy storage: An alternative to solar fuels. J. Mater. Chem. A 4, 2766–2782 (2016).

[b11] Hodes G., Manassen J. & Cahen D. Photoelectrochemical energy conversion and storage using polycrystalline chalcogenide electrodes. Nature 261, 403–404 (1976).

[b12] Licht S., Hodes G., Tenne R. & Manassen J. A light-variation insensitive high efficiency solar cell. Nature 326, 863–864 (1987).

[b13] Licht S. & Manassen J. Thin film cadmium chalcogenide/aqueous polysulfide photoelectrochemical solar cells with in-situ tin storage. J. Electrochem. Soc. 134, 1064–1070 (1987).

[b14] Hauch A., Georg A., Opara Krašovec U. & Orel B. Photovoltaically self-charging battery. J. Electrochem. Soc. 149, A1208–A1211 (2002).

[b15] Saito Y. . Energy-storable dye-sensitized solar cells with interdigitated nafion/polypyrrole-Pt comb-like electrodes. Chem. Lett. 39, 488–489 (2010).

[b16] Nagai H. & Segawa H. Energy-storable dye-sensitized solar cell with a polypyrrole electrode. Chem. Commun. 10, 974–975 (2004). 10.1039/b400439f 15069501

[b17] Liu P. . A solar rechargeable flow battery based on photoregeneration of two soluble redox couples. ChemSusChem 6, 802–806 (2013). 23559517 10.1002/cssc.201200962

[b18] Yan N. F., Li G. R. & Gao X. P. Solar rechargeable redox flow battery based on Li_2_WO_4_/LiI couples in dual-phase electrolytes. J. Mater. Chem. A 1, 7012–7015 (2013).

[b19] Yan N. F., Li G. R. & Gao X. P. Electroactive organic compounds as anode-active materials for solar rechargeable redox flow battery in dual-phase electrolytes. J. Electrochem. Soc. 161, A736–A741 (2014).

[b20] Kim W., Seok T. & Choi W. Nafion layer-enhanced photosynthetic conversion of CO_2_ into hydrocarbons on TiO_2_ nanoparticles. Energy Environ. Sci. 5, 6066–6070 (2012).

[b21] Kim J., Lee C. W. & Choi W. Platinized WO_3_ as an environmental photocatalyst that generates OH radicals under visible light. Environ. Sci. Technol. 44, 6849–6854 (2010). 20698551 10.1021/es101981r

[b22] Higashimoto S. . Photocharge-discharge behaviors of hybrid WO_3_/TiO_2_ film electrodes. J. Electrochem. Soc. 154, F48–F54 (2007).

[b23] Mi Q. . Photoelectrochemical oxidation of anions by WO_3_ in aqueous and nonaqueous electrolytes. Energy Environ. Sci. 6, 2646–2653 (2013).

[b24] Lee S. H. . Crystalline WO_3_ nanoparticles for highly improved electrochromic applications. Adv. Mater. 18, 763–766 (2006).

[b25] Saito Y., Uchida S., Kubo T. & Segawa H. Surface-oxidized tungsten for energy-storable dye-sensitized solar cells. Thin Solid Films 518, 3033–3036 (2010).

[b26] Kim G. & Choi W. Charge-transfer surface complex of EDTA-TiO_2_ and its effect on photocatalysis under visible light. Appl. Catal. B. 100, 77–83 (2010).

[b27] Kim H.-i. . Harnessing low energy photon (635 nm) for the production of H_2_O_2_ using upconversion nanohybrid photocatalysts, Energy Environ. Sci. 9, 1063–1073 (2016).

