# Supplementary material for: Solar-rechargeable battery based on photoelectrochemical water oxidation: Solar water battery
Source: Sci Rep. 2016 Sep 15;6:33400. doi: 10.1038/srep33400 (PMC5024119; doi:10.1038/srep33400)
Supplement: Supplementary Information [file srep33400-s1.doc]

Supplementary Information

**Solar-rechargeable battery based on photoelectrochemical water oxidation: Solar water battery**

Gonu Kim, Misol Oh and Yiseul Park*

*Corresponding author. Tel.: +82-53-785-3547; Fax: +82-53-785-3439

E-mail address: dewpark@dgist.ac.kr (Y. Park)

Division of Nano and Energy Convergence Research, Deagu Gyeongbuk Institute of Science & Technology (DGIST), 333, Techno Jungang Daero, Hyeonpung-myeon, Dalseong-gun, Daegu 42988, Korea


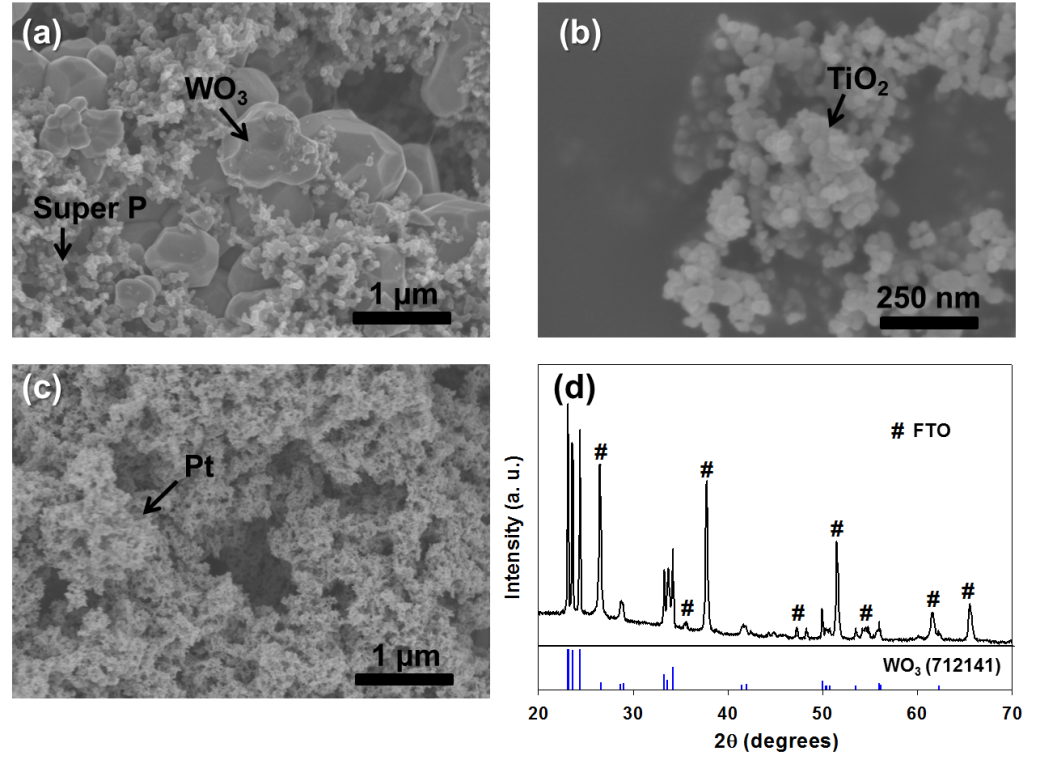


**Supplementary Figure 1.** FE-SEM images of (a) WO_3_ (b) TiO_2_ and (c) Pt on FTO electrodes. (d) XRD pattern of the WO_3_ on FTO electrode (Ref #712141)


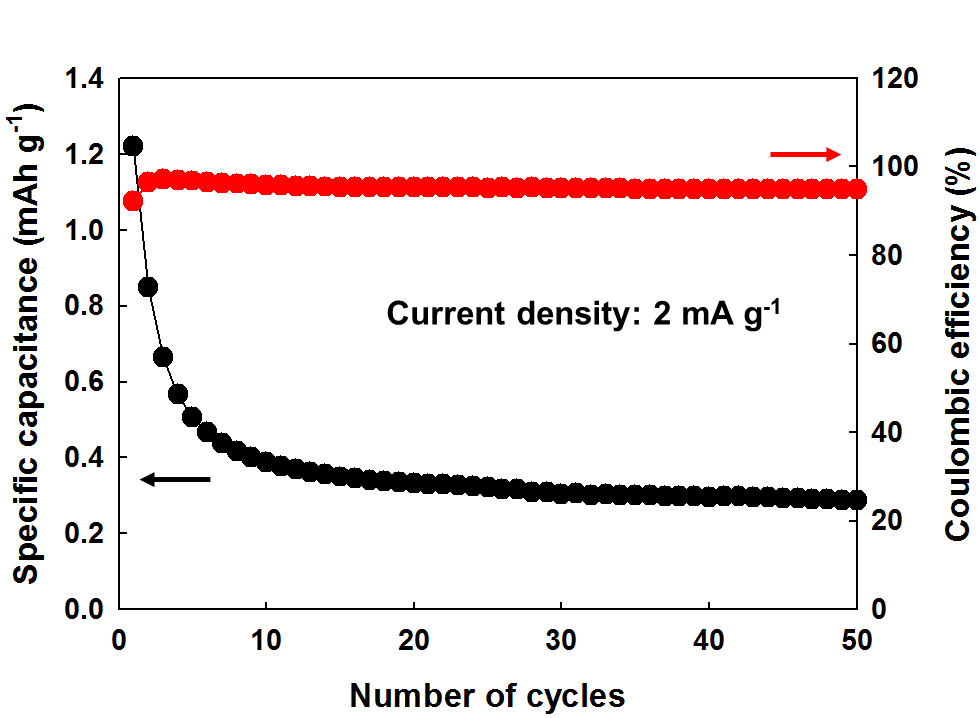


**Supplementary Figure 2.** Cycle performance of WO_3_ electrode for electrochemical charge/discharge at current density of 2 mA g^-1^ in dark condition. (Cutoff voltage –1.1 V, working electrode: WO_3_ electrode, counter, reference electrode: Pt electrode).


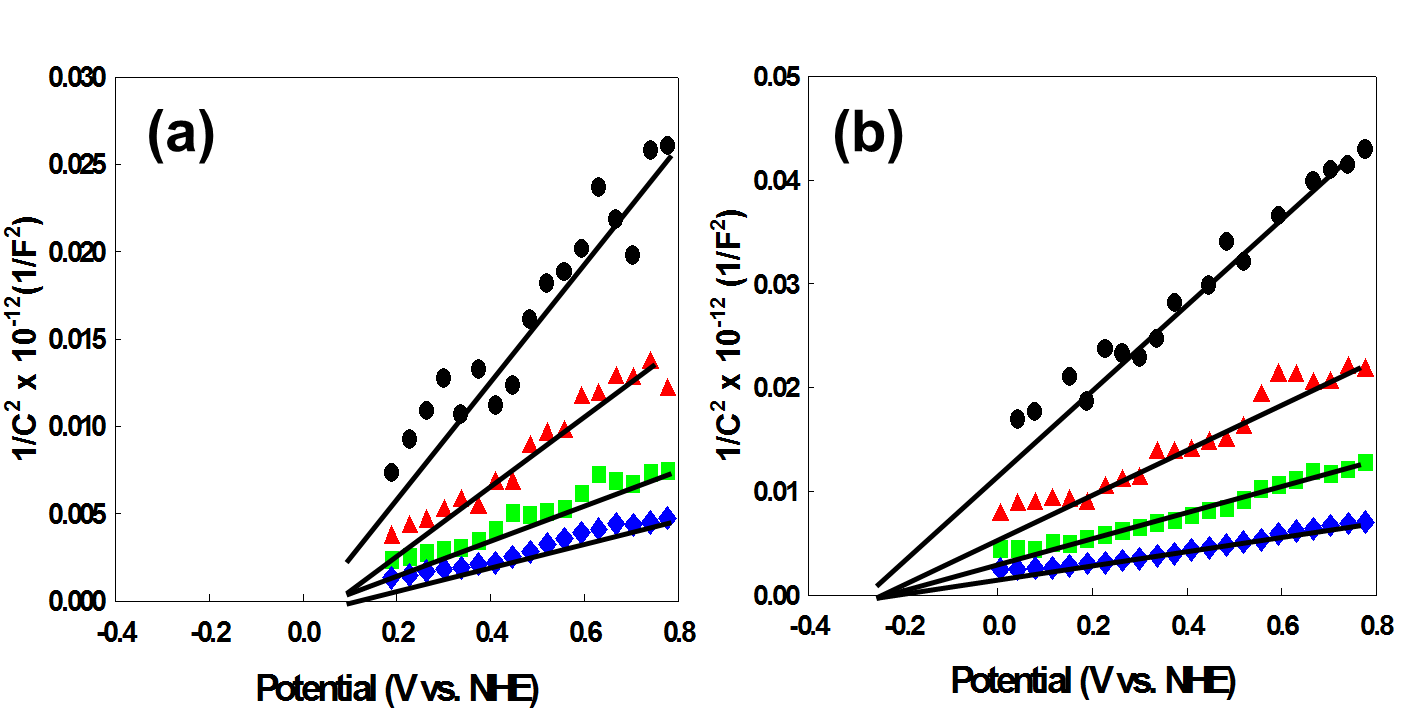


**Supplementary Figure 3.** Mott-Schottky plots of WO_3_ electrode (a) before photocharge and (b) after 16 h of photocharge (circles: 10.020 kHz, triangles: 4.641 kHz, squares: 2.156 kHz, and diamonds: 1.000 kHz).


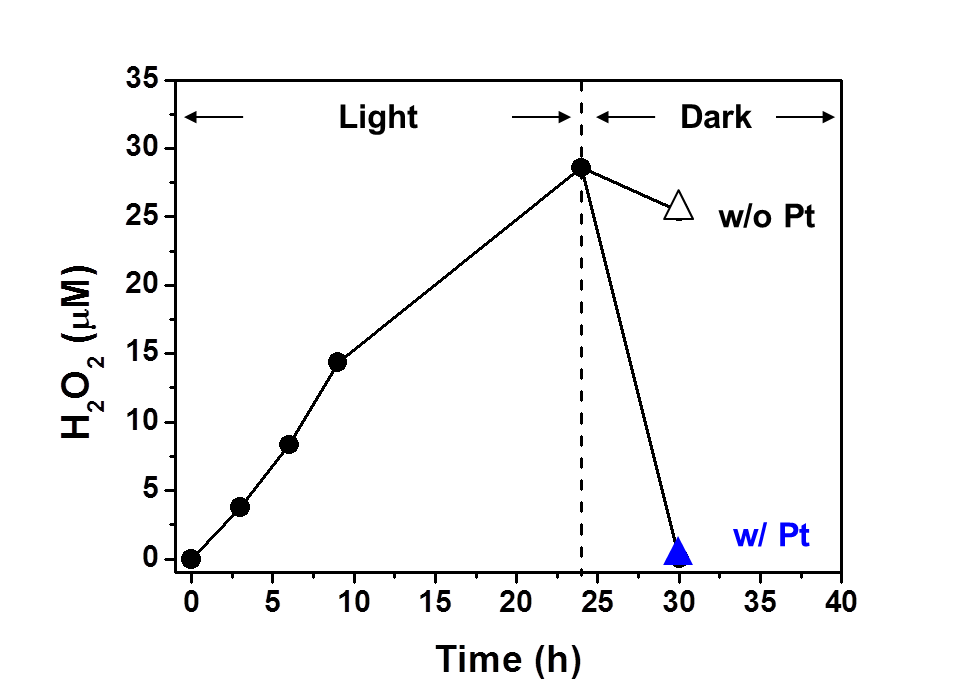


**Supplementary Figure 4.** H_2_O_2_ generation of TiO_2_-PE during the photocharging in the absence of Pt-CE. And H_2_O_2_ decomposition by adding Pt electrode in the solution after the photocharging. (10 mM Li_2_SO_4_, pH 3)


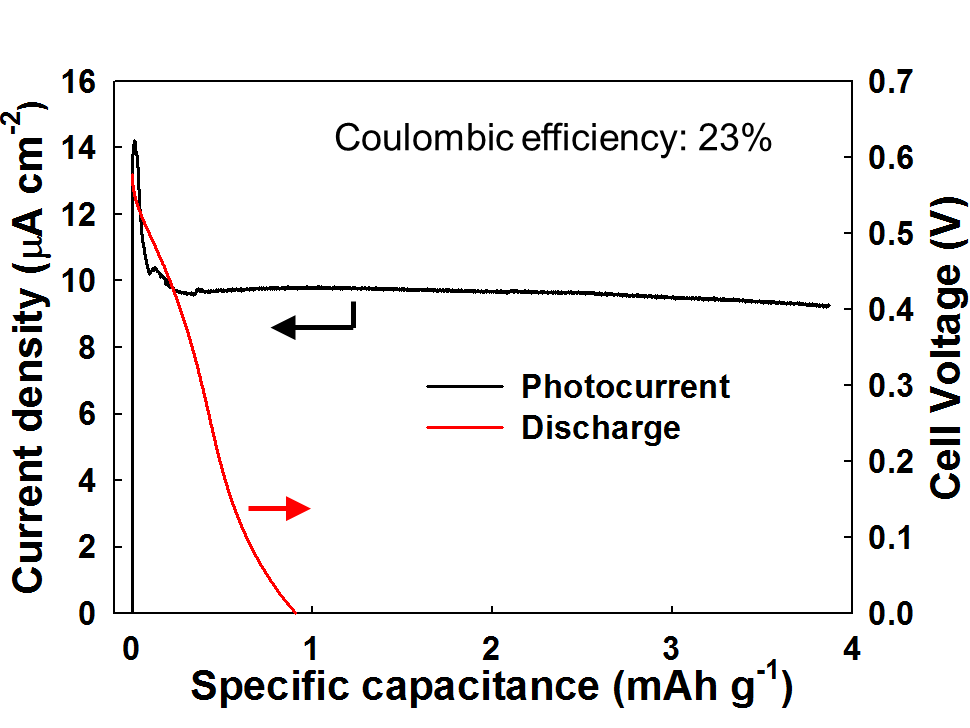


**Supplementary Figure 5.** Photocurrent generation and galvanostatic discharge curve of solar water battery when both anode and cathode parts are in anoxic condition.


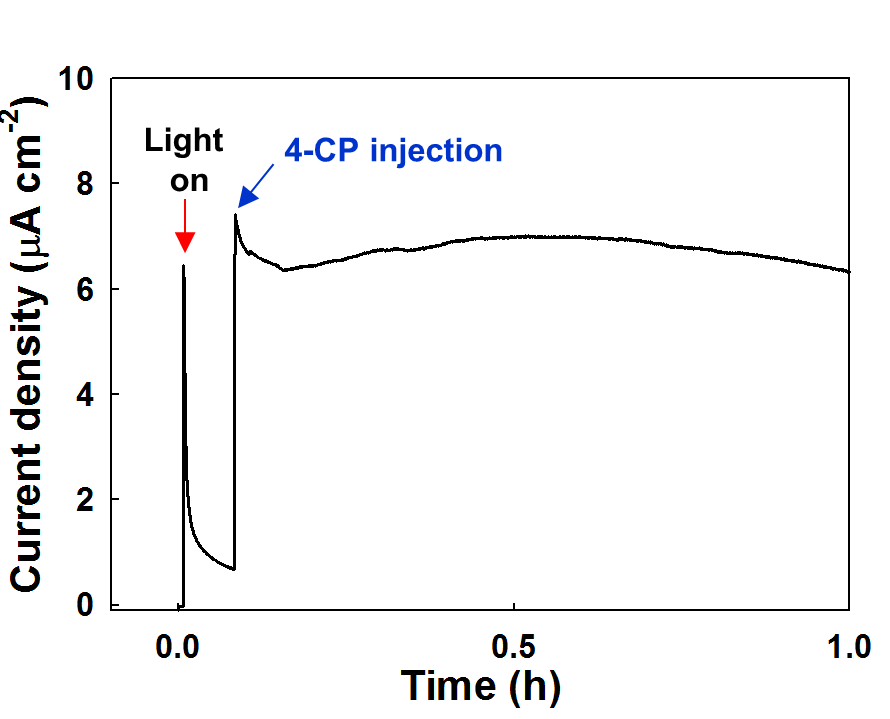


**Supplementary Figure 6.** Time profile of photocurrent generation. (all conditions are the same as those in Fig. 1 except for presence of 10 mM of NaF in cathode part).

In addition to photocharging based on water oxidation, the solar water battery can be applied to the removal of pollutants. Because cathode part of the solar water battery is opened to air, pollutants can be easily added to the cathode part and used as electron source for photocharge. For example, Supplementary Fig. 6 shows change in the photocurrent by the addition of 4-Chlorophenol (4-CP): a nonbiodegradable pollutant. This experiment (simultaneous production of electricity and degradation of organic compounds) was conducted in the presence of 10 mM of NaF to modify surface of TiO_2_ with fluorides. The surface fluorination of TiO_2_ is effective to hinder charge recombination between photo-excited electrons in TiO_2_ and oxidized 4-CP.^1,2^ After 5 min of light irradiation, we intentionally added 4-CP to the cathode part to make final concentration of 4-CP to be 100 µM. And after the injection of 4-CP, the photocurrent was sharply increased implying immediate degradation of 4-CP. This result clearly shows the addition of 4-CP induces the increase in photocurrent, and so the solar water battery have great potential be used for the removal of pollutants and generation of electricity simultaneously. More detailed research for development of such pollutant degradable solar water battery is currently under investigation.

**References**

1. Kim, J.*,* Monllor-Satoca, D. & Choi, W*.* Simultaneous production of hydrogen with the degradation of organic pollutants using TiO_2_ photocatalyst modified with dual surface components. *Energy Environ. Sci.* **5**, 7647-7656 (2012).
2. Kim, J., Lee, J. & Choi, W. Synergic effect of simultaneous fluorination and platinization of TiO_2_ surface on anoxic photocatalytic degradation of organic compounds. *Chem. Commun.* 756-758 (2008)
